# Identifying a nuclear passport for HIV

**DOI:** 10.7554/eLife.45580

**Published:** 2019-03-05

**Authors:** Lorena Zuliani-Alvarez, Greg J Towers

**Affiliations:** Division of Infection and ImmunityUniversity College LondonLondonUnited Kingdom

**Keywords:** Human immunodeficiency virus, CPSF6, macrophages, capsid, nuclear pore complex, nuclear import, Human, Virus

## Abstract

Identification of a protein that pulls HIV into the nucleus explains a key step in HIV infection.

**Related research article** Bejarano DA, Peng K, Laketa V, Börner K, Jost KL, Lucic B, Glass B, Lusic M, Müller B, Kräusslich HG. 2019. HIV-1 nuclear import in macrophages is regulated by CPSF6-capsid interactions at the nuclear pore complex. *eLife* **8**:e41800. doi: 10.7554/eLife.41800

Cells have many ways to fight pathogens, but they still manage to evade our defenses. HIV, for example, is able to enter a cell, cross the cytoplasm, and then pass through the nuclear pore complex to reach the cell nucleus. The DNA within the virus is surrounded by a capsid that protects against defense mechanisms of the host, such as nucleic acid sensors and nucleases (Yan et al., 2010; Jacques et al., 2016; Francis and Melikyan, 2018; Burdick et al., 2017; Rasaiyaah et al., 2013).

Previously, it was thought that the capsid cannot enter the nucleus, but demonstration that the capsid depends on a nuclear protein called CPSF6 suggested that it might (Price et al., 2014; Lee et al., 2010). Now, in eLife, Hans-Georg Kräusslich and colleagues – including David Alejandro Bejarano and Ke Peng as joint first authors – report new insights into the role of CPSF6 and the entry of the capsid into the nucleus (Bejarano et al., 2019). The researchers – who are based at the University of Heidelberg and the Chinese Academy of Sciences – tagged HIV molecules with fluorescent markers and observed how HIV infected macrophages. The capsids and CPSF6 proteins were labeled with antibodies.

Bejarano et al. found that, contrary to previous assumptions, CPSF6 is essential for HIV to travel through the nuclear pore into the nucleus of macrophages. They also found that CPSF6 associated with capsids inside the nucleus, suggesting that intact capsids may cross the nuclear pore. When CPSF6 was depleted, or could no longer bind to the capsid, the virus accumulated outside the nucleus. This indicates a key role for CPSF6 in viral nuclear entry. However, similar to previous experiments, removing CPSF6 only modestly reduced the infection levels in macrophages because the capsids used the remaining low CPSF6 levels.

It is known that the capsid also binds a nuclear pore protein called Nup153 (Price et al., 2014). Bejarano et al. suggest that the capsid first interacts with Nup153 inside the nuclear pore complex; Nup153 is then displaced by nuclear CPSF6, which helps to pull the capsid inside the nucleus (Figure 1). They also propose an intriguing model to explain how the viral DNA could integrate into host DNA in the absence of CPSF6. In line with the hypotheses proposed in other studies (Achuthan et al., 2018; Schaller et al., 2011; Sowd et al., 2016), Bejarano et al. suggest that, in the absence of CPSF6, the capsid pokes through the nuclear pore complex enough for the integration of DNA to take place at the periphery of the nucleus.

**Figure 1.**
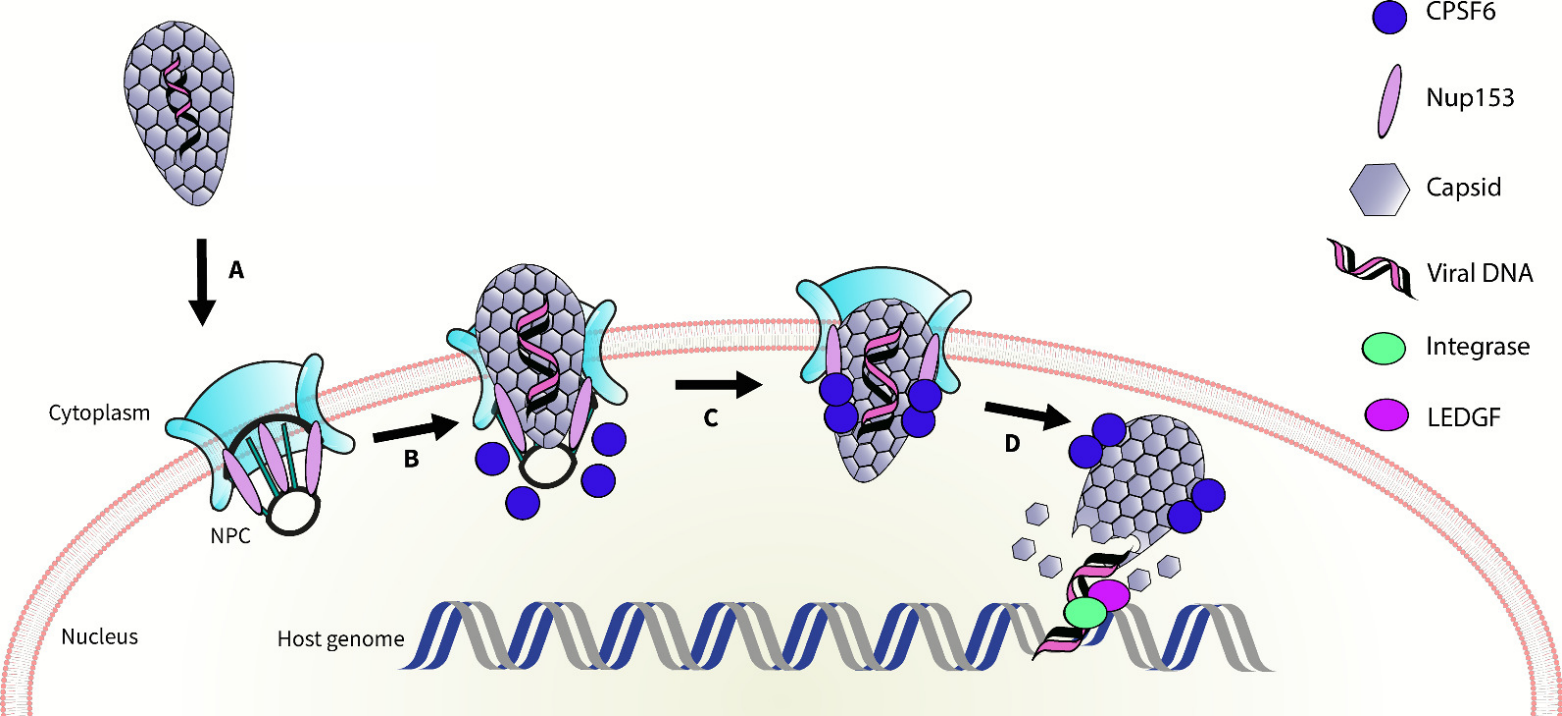
Schematic of HIV entering the nucleus. The genetic material of HIV is surrounded by a capsid (grey honeycomb structure). Previously, it was thought that the virus sheds this capsid before it enters the nucleus. Bejarano et al. now show that, in macrophages, the virus retains its capsid while entering the nucleus, through the nuclear pore complex (NPC; turquoise), Two proteins, CPSF6 (blue circle), and Nup153 (lilac oval) control this process. First, Nup153 binds to the capsid as it associates with the pore complex. CPSF6 then binds to the same region of the capsid, displacing the Nup153, and helping the capsid enter the nucleus. Finally, the capsid ‘uncoats’ and the viral DNA integrates into the host DNA with the help of an enzyme called integrase (green oval) and a protein called LEDGF (purple oval).

Two key questions remain. First, why is CPSF6 required for replication in macrophages but not in cell lines? One reason for this might be that, in the absence of binding to a cofactor such as CPSF6, HIV-1 triggers the production of interferon in macrophages (Rasaiyaah et al., 2013). We can think of this in terms of cell lines 'licensing' otherwise 'illegal' behaviors by viruses. For example, capsid uncoating in the cytoplasm is 'illegal' in macrophages: that is, it does not lead to infection. However, such behaviour is licensed in cell lines and results in infection. This might be because, in macrophages, interferon suppresses mutant HIV-1 infection and uncoated cytosolic capsids are degraded. In cell lines, on the other hand, these 'illegal' viral behaviours are allowed and the virus can replicate.

Second, what is the role of CPSF6 in T cells (the immune cells that are the main target of HIV)? Given their small size and minimal cytoplasm, T cells are less suitable for microscopy, although nuclear transport can be studied in them (Achuthan et al., 2018). A better understanding of how HIV replicates in T cells, and where capsid uncoating occurs, may reveal fundamental differences in infection mechanisms between T cells and macrophages. In turn, this may help us understand the relative importance of HIV replication in macrophages and T cells in vivo.

This elegant study is a real technical tour de force, which has overcome significant experimental challenges to answer key questions in the field. As the techniques used by Bejarano et al. become widely adopted, we look forward to rapid advances in our understanding of the basic biology of HIV that may ultimately lead to more effective ways to treat, and eventually cure, HIV and AIDS.
